# The Ratio of the Genome Two Functional Parts Activity as the Prime Cause of Aging

**DOI:** 10.3389/fragi.2020.608076

**Published:** 2020-11-16

**Authors:** Lev Salnikov, Mamuka G. Baramiya

**Affiliations:** ^1^ SibEnzyme US LLC, West Roxbury, MA, United States; ^2^ AntiCancer, Inc., San Diego, CA, United States

**Keywords:** aging, senescence, housekeeping genes, integrative genes, multicellularity, carcinogenesis, epigenetic, rejuvenation

## Abstract

The *metazoan* genome composes of sets of housekeeping genes (HG) for fundamental cellular autonomous processes and integrative genes (IntG) that provide integrative functions and form the body as an integrated whole. The main paradigm for multicellularity development which has been improved in evolution, is the submission of the cellular autonomy to the interests of the integrated whole. Permanent increase of the “functional tax” of IntG-genome (IntG-shift) and epigenetic restriction of autonomy in phylogenesis/ontogenesis is the essence and root cause of aging, inherent in the very nature of highly integrated multicellularity. The regulation of the balance shift toward HG can be managed to eliminate aging and avoid carcinogenesis, which is only due to the irreversibility of this shift. Here we propose the criterion for measuring the functional and biological age of cells and the body as a whole for assessing the effectiveness of any type of palliative geroprotective or radical anti-aging intervention.

*“There are good reasons to suspect that heterogeneity (i.e., variability within any given set of samples) is an essential characteristic of organic life. This idea differs widely from the traditional view that heterogeneity is only a nuisance that is to be circumvented or otherwise eliminated.” Elsasser WM* ( Elsasser, 1984).

## Introduction

At present, there is no single, commonly accepted theory of aging. All main ideas about the essence of this biological phenomenon can be divided into two large groups. There are two main scientific hypotheses of the understanding of the essence and causes of the aging of multicellular organisms ( *metazoans)*—“initial imperfection” and the existence of an “aging program.” Research in these areas is ongoing.

All attempts to explain the phenomenon of aging based on the postulate of “deterioration” as a consequence of “primordial imperfection,” face the need to find one main link that could stop the aging processes. Today, the role of such a link is claimed by the “inability” of the cell itself to fully restore damage to its genome with aging. This conclusion follows from experimental data ( Gorbunova et al., 2007). An increase in non-repairable damage of cellular DNA in senescent cells, compared to young cells, allows one rise to the conclusion that this is the cause of aging. However here the cause of the phenomenon perhaps replaced by the consequence since “after that” does not always mean “because of that.”

A second postulate—specific “aging program” for *metazoans*, based on the evolutionary necessity of aging as a mechanism for changing generations, suggests the possibility to manage it, or to turn it off, which in fact, is the ultimate goal ( Kirkwood and Holliday, 1979; Kirkwood and Austad, 2000).However, such a program, allowing extending the lifespan several times more than on average, has not yet been described.

Therefore, there is still no hypothesis or theory of underlying mechanism of aging that could be verified experimentally. It is possible to study deeper and deeper levels of organization of biological structures - from molecular to quantum, but following this logic of analysis it is impossible to find an answer to the main question about the root cause of aging.

### Functional Parts of *Metazoans* Genome

In order to understand how the mechanisms of aging work in metazoans, it is necessary to determine what the object we are actually studying. Naturally, such an object is the multicellularity *per se*. Considering it from the point of view of evolution and ontogenesis, it is necessary to note that the genome of all multicellular organisms can be partitioned in two parts, different in their function. Thus, in the course of ontogenesis multicellularity is not built based on all elements of, but only part of the genome, which changed in the course of evolution. The other part of the genome remained virtually unchanged, constantly ensuring the viability of the cells themselves. These genes are similar to most of the genes in unicellular organisms.

Thus, as a prerequisite for the development of the body, the DNA of all its cells contain two *functionally* independent parts. One part of the genome that provides for the internal needs of any cell, or housekeeping genes (HG) ( Tagle et al., 1988; Lenhard et al., 2003; Wray et al., 2003; Farré et al., 2007). The other functional part of the cellular genome is the genes, that provide the *integrative function*, or genes responsible for all specialized structures produced by cells in the course of differentiation and creating the organism as an integrated whole (IntG). As a result, all multicellular organisms contain two causally closed, or autopoietic ( Maturana and Varela, 1980) systems, based on the HG and IntG parts of the genome. It should be noted here that by HG we mean genes that work exclusively for autonomous intracellular needs, but not related to integrative functions of the cell specialization.

From our point of view, multicellular organism is a way of existence of a cell colony, in the course of individual development in which (ontogenesis) an independent system composing a collective cell symbiote, which is necessary for the existence of the colony itself. At the same time, in the course of ontogenesis, the symbiotic part becomes parasitic, leading to the exhaustion of resources and aging in the broadest meaning of this concept ( Salnikov, 2012). Let us consider how the total expression of IntG and HG changes in the cells of the body during its development.

### Synchronous Development of IntG and HG Parts and Correlation of Their Activity During Ontogenesis

During ontogenesis, starting from its earliest stages, there are correlated changes of activity in certain parts of IntG and HG genomes. To start histogenesis and organogenesis, the activity of a certain part of the IntG is required, which is accompanied by epigenetic downregulation of HG, part responsible for complete autonomy (toti and pluripotency). Such a “chain reaction” of IntG and HG activity, not only allows the cells (and tissues and organs formed by them) to reach the necessary level of differentiation and specialization, but also limits their autonomy.

Functional activity of HG is maintained at sufficiently constant level during the first half of ontogenesis. This is necessary to create the “framework” of the body. One can see a relative decrease in the level of HG activity only in its second half, which is a consequence of the functional predominance of the IntG-genome, which begins closer to the middle of ontogenesis. This functional part of the genome reaches its maximum activity, required to complete the ontogenesis (or fill the body’s “framework”) later than HG. For both individual cells and the body, as a whole, there is a functional optimum zone. This is a state, in which the programmed level of development is accompanied by the lowest metabolic costs for its maintenance. For both individual cells and the whole organism, this occurs at different times. The organism’s optimum is always reached later than the cellular one. Such disparity naturally leads to “pushing out” the cells of an organism from an optimum zone, leading to a similar situation for an organism as a whole.

Thus, we speak about the system-forming principle for highly specialized multicellularity on which it is built and which determines the course of multicellular organism development and constitutes the essence of aging.

Our proposed approach allows us to establish the main criterion to measure functional age in single cells, which defines its functional, and then its biological aging, as well as the aging of the organism as a *whole.* In fact, this is nothing more than a zero-sum game in conditions of limited resources. In other words, the impossibility of complete (quantitative and qualitative) replacement of system components transforms their functional part from a cell symbiote into a cell parasite. This main consequence of the ontogenesis, inevitably leads to the degradation resulting in organism aging.

The total housekeeping genome (THG) consists of an autonomizing HG (AHG) section, whose work (corresponding to the totipotency, pluripotency, and most likely to some extent multipotency of the embryo) is gradually and reversibly (epigenetically) blocked in early ontogenesis (starting from gastrulation) and growth HG (GHG), whose functioning increases in histogenesis and organogenesis and further during whole postnatal growth period and then is also gradually and reversibly blocked in postnatal ontogenesis ( THG= AHG+ GHG ). Ontogenesis conditionally includes several stages, carried out by different development modules, depending on the IntG/GHG ratio, which ultimately defines the functional and biological age of both cells and the organism as a whole:

1. Early prenatal stage (morphogenetic + growth module - IntG/THG ≪1 ).
2. Late prenatal stage (growth module - IntG/GHG ≪1 ).
3. Postnatal stage of growth (growth module – IntG/GHG < 1 ).
4. Compensated (balanced) development stage - (regeneration module - IntG/GHG < 1 ⇄ > 1 ).
5. Early decompensated (unbalanced) aging stage - (involutionary module - IntG/GHG > 1 ).
6. Late decompensated (unbalanced) aging stage - (involutionary module - IntG/GHG ≫ 1 ).

Under conditions of constant exposure to exogenous damaging factors, when metabolism of by-products and endogenous damaging factors are partially or completely impaired, a “shift” toward repair and proliferative processes, or a GHG-shift is required for adequate compensation of functions (or, otherwise, successful functioning of the IntG-genome itself), which ensures restoration of tissue and function deficiency. This is how the body responds to damage caused by various stressors at the various stages of growth and development (and with varying degrees of efficiency at different stages of compensated aging). On the contrary, at the stage of decompensated aging, cells respond to such damage by permanent cell cycle arrest (up to complete stoppage), against the background of a steady decrease in the efficiency of reparative processes ( Gorbunova et al., 2007; Childs et al., 2015; Lagunas-Rangel and Bermúdez-Cruz, 2019).

However, in the decompensated aging stage a greater flexibility is needed to eliminate the steadily growing amortization load than it can carry out within the state of relatively rigid specific determination and the level of differentiation that determines it, which is characteristic for the definitive tissues. In other words, a low amplitude and high frequency GHG-shift or regeneration modules can no longer handle adequate quantitative and qualitative replacement of lost tissues and impaired functions. To execute this task in late postnatal ontogenesis, a high amplitude THG (AHG + GHG)-shift is required, which is typical for early prenatal ontogenesis.

However, such a degree of autonomy in postnatal ontogenesis conflicts with the nature of highly organized/highly integrated multicellularity, of which the main principle of functioning is in strict subordination of constituent parts (cells) “freedom” to the integrative interests of the whole (paradoxically as a result, to the detriment of these interests). Instead, we get an increasing “shift” toward the elimination of damaged cells, with the increased prevalence of functional specialization (IntG-shift and involutionary module). Going deeper into this state leads to a narrowing of adaptive potential spectrum (to relative adaptive rigidity), a reduction of the self-renewal rate, an increase in the number of mutations, the appearance of non-self-reproducing units in self-reproducing systems, a decrease in the resistance of cells and tissues to exogenous harmful factors and coarse qualitative changes in regenerative processes.

As a result, perished structures are replaced by non-specific, deficient structures, metabolic provision of hyperfunctioning of remaining rigged specialized structures becomes unattainable, their functional insufficiency is increased, absolute deficiency of tissues and functions reaches critical level, processes of endogenous intoxication are potentiated, which makes the organism even more vulnerable to exogenous factors. In the long run there is a paradoxical situation, when limitating autonomy in favor of integration/functional specialization causes damage the latter ( Baramiya, 1988; Baramiya, 2000). Therefore, eventually aging at the whole organism level is predetermined by senescence at the cellular level and it is cellular senescence that is the basic aging mechanism ( Borghesan et al., 2020).

Consequently, a permanent increase of the “ *functional tax*” of the IntG-genome (IntG-shift) accompanied by simultaneous epigenetic restriction of autonomy (freedom of maneuver) in phylogenesis and ontogenesis is the essence and prime cause of aging inherented in the very nature of highly integrated multicellularity.

We argue that aging and death are the price for such multicellularity. It means that aging is not some stand-alone program, but also not a stochastic accumulation of random errors. Aging is an integral part of the development program (ontogenesis) of highly specialized multicellularity, carried out through epigenetic blocking of autonomous cellular annulling of metabolic and genetic “amortization cargo” (self-rejuvenation) in favor of integrative status quo, which gradually damages this status itself. The rest is secondary and without understanding of this, there is only random walking in the maze of hysteron-proteron.

Importantly that the main feature of ontogenesis in highly specialized *metazoans* is its unidirectionality and ubiquity. Such pervasive unidirectionality from autonomy to rigid specialization and death is impossible to overcome principally on the non-systemic level. In other words, such feature do not allow modifying ontogenesis through its isolated links or mechanisms. To override the program is possible only on the system level by another program - the program of permanent re-ontogenesis/re –morphogenesis ( Baramiya and Baranov, 2020; Baramiya et al., 2020).

### Rejuvenation as a Fine Line Between Cancer and Aging

Based on the above, the success criterion for any healthspan intervention outcome will be the recreation of the HGH-shift profile or, in the other words, the IntG/GHG ≤1  profile. The same criterion for successful strategic anti-aging (aging elimination) interventions will be the recreation of the THG-shift profile or, in the other words, the IntG/THG ≪1  profile.

In other words, the scale of intervention is determined by the extent of decline of the “burdens” of integrative functions and the success of intervention is determined by the ability not to “roll down” into irreversible autonomization - carcinogenesis.

Schematically, the status quo can be presented as: Cancer “Death Road” ← IntG/GHG ≪1 ↮ IntG/GHG ≫ 1 → Senescence “Death Road.”

Solution of the problem is in the “looping” of processes and can be presented as:

Rejuvenation ← IntG/THG ≪1 ⇄ IntG/GHG > 1   → Senescence

It is known that most diseases, that medicine faces are the result of age-related changes in the body, which forces modern medicine try to “treat aging.” Any pathological process is to some extent irreversible (and therefore progressing at one or another rate) homeostasis deviation, which is normally regulated by many feedback mechanisms/signal pathways and a huge network of their interactions and interconnections. Importantly that it is namely irreversibility of the deviation that is the pathology, and to a lesser extent the amplitude (within vitality) of that deviation. For example—pregnancy is also a very significant deviation, but substantial results can sometimes not be achieved without a significant deviation. Any intervention is aimed to bring homeostasis to a state of adequate dynamic balance. Optimally - to a self-maintenance balance. However, we do not always know which stage of the decompensated deviation our exposure belongs and, more importantly, how the impact on a certain mechanism at this stage will affect others and what it will eventually lead to.

Speaking about the necessity of homeostasis deviation in the application to the self-renewal processes and the necessity of a significant degree of this deviation toward autonomization (with temporary “damage” to the integrative processes) in order to achieve permanent re-ontogenesis, it is important to remember, that “In a complex hierarchical system, the growth of diversity at the upper level is provided by the restriction of diversity at the previous levels, and vice versa, the growth of diversity at the lower level [of the hierarchy] destroys the upper level of the organization” ( Nazaretyan, 2004). However, it should be clarified, and it is fundamental that we should not just talk about the growth of diversity on the lower hierarchies, but about the limitlessness, irreversibility and uncontrollability of this growth, because they are the ones that convert this deviation in disintegrating process (malignant growth). On the contrary, in case of reversibility/manageability this deviation radically renews, making the integrated (and vulnerable) system unlimitedly self-renewable and stable, as detailed in our previous publications ( Baramiya and Baranov, 2020; Baramiya et al., 2020).

## Discussion

The point of view, based on genome division into two functional parts - HG and IntG, leads us to the conclusion, that the very way of organizing highly integrated multicellularity result in cellular senescence, whole organism aging and is its root cause.

By measuring IntG/GHG ratio, one may be able to make substantiated assessment of functional and biological age from the developmental biology point of view and corresponding efficacy of any type of palliative geroprotective (healthspan) or radical anti-aging (reprogramming and aging elimination) intervention may be possible.

This approach allows us to offer an evolutionarily justified strategy for aging elimination and a method for achieving this goal.

## Author Contributions

LS has proposed the general concepts of functional genome partition and the role of ratio of the genome two functional parts activity in ontogenesis and for determine functional and biological age.

MB has introduced the separation of total housekeeping genome into functional parts, periodization of ontogenesis based on IntG/GHG ratio, the concepts of IntG-, GHG- and THG-shifts and the looped ontogenesis as an alternative for unidirectional ontogenesis for aging termination through permanent re-morphogenesis.

The concept about ratio of the genome two functional parts activity as the prime cause of aging formulated jointly.

## Funding

The authors have no relevant affiliations or financial involvement with any organization or entity with a financial interest in or financial conﬂict with the subject matter or materials discussed in the manuscript. This includes employment, consultancies, honoraria, stock ownership or options, expert testimony, grants or patents received or pending, or royalties. No writing assistance was utilized in the production of this manuscript.

## Definitions

Regenerative module—a set of restoration processes in tissues within the differentiated status.

Morphogenetic module—a set of restoration processes in tissues with recapitulation of the early stages of ontogenesis and the appearance of pluripotent germ-type blastema cells characteristic of the period of embryonic development and epimorphic regeneration.

Involutive module—a set of compensation processes in tissues within the involutive stage of development.

## Conflict of Interest

LS was employed by the company SibEnzyme US LLC., and MB was employed by AntiCancer Inc.

The remaining author declares that the research was conducted in the absence of any commercial or financial relationships that could be construed as a potential conflict of interest.

## Abbreviations

HG, housekeeping genes; AHG, autonomizing housekeeping genes; GHG, growth housekeeping genes; THG, total housekeeping genome; IntG, integrative genes.
